# Efficacy of respiratory rehabilitation in patients with COVID-19: a retrospective study

**DOI:** 10.1186/s12890-024-02969-z

**Published:** 2024-03-26

**Authors:** Zhiyou Zhang, Congcong Wang, Zhendong Li, Yueyang Liu, Yutong Nie, Jianwei Zhang, Dawei Li

**Affiliations:** Department of Neurorehabilitation, Shengli Oilfield Central Hospital, No. 31 Jinan Road, 257000 Dongying, Shandong China

**Keywords:** Airway clearance technology, Prone position ventilation, COVID-19, Rehabilitation effect

## Abstract

**Objective:**

The coronavirus disease 2019 (COVID-19) pandemic has resulted in millions of confirmed cases and deaths globally. The purpose of this study was to investigate the therapeutic effect of airway clearance technology combined with prone ventilation on patients infected with COVID-19.

**Methods:**

38 patients with COVID-19 (severe) who were treated in the intensive rehabilitation group of Shengli Oilfield Central Hospital. They were randomly divided into a control group and an observation group. The control group received prone position ventilation intervention, and the observation group received airway clearance technology combined with prone position ventilation intervention. The changes of oxygen and index, procalcitonin (PCT), interleukin-6 (IL-6) and chest X-ray image indexes were compared between the two groups.

**Result:**

There was no significant difference in age, gender and other general data between the control group and the observation group. The results showed that oxygen index, PCT, IL-6 and chest X-ray image index in the observation group were better than that indexes in the control group.

**Conclusion:**

Airway clearance technology combined with prone ventilation intervention in patients with COVID-19 can improve the total effective rate and oxygenation index, improve the inflammatory indicators and respiratory function of patients. And it may be widely promoted and used in the treatment of patients with COVID-19 (severe).

## Introduction

Coronavirus disease 2019 (COVID-19) caused by severe acute respiratory syndrome Coronavirus 2 (SARS-CoV-2) has seriously affected human life and health [1, 2]. Some patients infected with the novel coronavirus have pulmonary fibrosis [3], and even develop into severe patients requiring intensive care unit (ICU) treatment [4]. The patient developed dyspnea and hypoxemia one week after onset. Severe cases may develop acute respiratory distress syndrome, septic shock, metabolic acidosis, and coagulation dysfunction [5–7]. Even patients with more serious dysfunction, such as changes in lung function, mental and cognitive function decline, motor function decline and other problems [8]. Indirect braking such as bed rest in the care unit and the use of sedatives led to disuse atrophy of the patient respiratory muscle, prolonged mechanical ventilation time, reduced respiratory function, and further aggravated pulmonary infection [9].Therefore, improving respiratory symptoms and optimizing respiratory function is one of the important goals of effective early rehabilitation.

In the latest autopsy results of COVID-19 patients, there are many sticky secretions in the airways [10]. Therefore, early rehabilitation intervention, effective sputum excretion, and increasing patients’ airway clearance ability are also particularly important in the treatment process. Prone position ventilation refers to mechanical ventilation with the patient lying on the stomach that improves ventilation and oxygenation by reducing lung compression and improving lung perfusion [11–13]. Therefore, we speculate that prone ventilation may improve the recovery effect of COVID-19 patients.

This study mainly carried out airway clearance technology combined with prone ventilation for COVID-19 patients (severe). At the same time, the rehabilitation effect and prognosis of patients with these two techniques were discussed.

## Materials and methods

### Patients’ inclusion

General data A total of 38 patients with severe COVID-19 (severe) who were treated in the severe rehabilitation group of Shengli Oilfield Central Hospital from December 2022 to February 2023 were selected as the study objects. And randomly divided into control group and observation group. In the control group, there were 11 males and 8 females, aged 55–81 years, with an average age of (73.8 ± 5.9) years. In the observation group, there were 13 males and 6 females, ranging in age from 47 to 80 years, with an average age of (72.0 ± 9.8) years. There was no significant difference in age, gender and other general data between the two groups (*P* > 0.05), which could be compared.

### Inclusion criteria

Inclusion criteria: [1] All of them meet the heavy diagnostic criteria for clinical classification of the “COVID-19 Diagnosis and Treatment Protocol (Trial 10th edition)”; [2] Shortness of breath, RR > 30 times/min; [3] In the resting state, the oxygen saturation of the finger is < 93%; [4] Arterial partial oxygen pressure (Pa02)/ oxygen absorption concentration (Fi02) < 300mmHg(1mmHg = 0.133 kPa), high altitude (elevation over 1000 m) areas should be corrected according to the following formula: Pa02/Fi02x [760/atmospheric pressure (mmHg)]; [5] The patient’s clinical symptoms worsened progressively, and lung imaging showed that the lesion progressed significantly > 50% within 24 to 48 h. And the hemodynamics have been relatively stable.

### Exclusion criteria

Exclusion criteria: [1] Patients with metabolic diseases; [2] Patients with congenital heart disease and pneumothorax; [3] There are injuries or wounds on the ventral body surface that affect the prone position; [4] Cervical vertebra and spine unstable fracture, need to be fixed; [5] Patients younger than 18 years and older than 80 years; [6] Pregnant women; [7] There is an obvious high risk of pulmonary embolism; [8] Active bleeding; [9] Unable to cooperate with treatment;

### Implementation method

#### Conventional therapy

Both the control group and the observation group had received vital signs monitoring to ensure adequate energy and nutrition intake, maintain internal environment stability, humidified oxygen therapy, sputum aspiration and other general conventional treatment and azvudine antiviral treatment. Patient with underlying diseases were given corresponding treatment, but the results of this experiment would not be affected.

#### Prone position ventilation intervention

Control group received prone position ventilation intervention: (1) Preoperative hemodynamics were evaluated. Confirm the safety of the pipeline, stop nasal feeding 2 h in advance, and pump back the stomach contents before operation to avoid reflux aspiration. (2) The patient is in a prone position to avoid pressure on the mouth, nose, chest, etc. The patient’s head can be tilted to one side, and the patient’s vital signs are monitored (3) At the same time, the patient’s psychological counseling. Prone position 2 times a day, 4–6 h each time, treatment for a week, if the patient has obvious complications promptly terminated.

#### Airway clearance technique combined with prone position ventilation therapy

Observation group received airway clearance technique combined with prone position ventilation treatment: (1) Rehabilitation therapists improve patients’ understanding of rehabilitation through communication and education to ensure patients’ peace and stability. (2) Prone position ventilation operation was the same as control group. Airway clearance techniques include: A: gravity was used to promote discharge of secretions in the head and foot position; B: Tap vibration was performed on the patient’s back with an auxiliary sputum discharge instrument to release mucus; C: Combined with the manual technique, shaking is performed in the exhalation phase; D: At the end of prone position, passive activities can be performed to loosen and ease the thorax of both ribs to increase the patient’s ventilation. Sputum negative pressure aspiration was performed after rehabilitation treatment. (3) Rehabilitation Psychology. Prone position 2 times a day, 4–6 h each time; At the same time, two airway clearance exercises were performed for 1 h each time, and the treatment was carried out for one week. During the rehabilitation process, pay attention to the patient’s facial expression, sputum and cough. If there is discomfort such as chest tightness, adjust the method or terminate it in time, and continue after the patient’s dyspnea improves.

Two groups of patients were treated for 1 week, and the changes of indicators were observed and recorded.

### Observational index

1. Oxygenation index and PaCO_2_: As a target in respiratory therapy, oxygenation index can reflect the body’s oxygenation status, and PaCO_2_ is an important indicator reflecting respiratory acid-base balance.
2. Procalcitonin (PCT): PCT is a protein that is specific to bacterial infections and can be used to judge the efficacy of the treatment of bacterial infections.
3. Interleukin-6 (IL-6): IL-6 is an important pro-inflammatory cytokine that is pleiotropic, it is induced by infection or tissue damage and rapidly triggers an acute response to minimize it [14]. IL-6 plays a role in triggering inflammatory responses and activating adaptive immunity against infection or injury [15].
4. Chest X-ray: Lung imaging manifestations include solid shadow, patch shadow, grid shadow, rope shadow, hilar mediastinum, pneumothorax, pleural effusion, pleural thickening, etc. The standard of lesion reduction was the reduction of lesion scope, number and density, and the lesion was transformed into a grid or fibrous strip shadow. The standard for the increase of lesions is that the scope of lung lesions increases, the structure is distorted, and the density increases, such as the ground glass density shadow changes into solid shadow [16]. Statistical improvement is' 1 ‘, no significant change or infection aggravation is' 2 ‘.

### Statistical analysis

SPSS 21.0 was used for data analysis. Normality test was performed for each group of data. The description of measurement data conforming to normal distribution was represented by mean ± standard deviation, comparison between the two groups was represented by t test, and the ratio of counting data (%) was represented by chi-square test. α = 0.05 was the test level, and *P* ≤ 0.05 was considered statistically significant.

## Results

### Comparison of oxygenation index and PaCO_2_ before and after treatment between the two groups

After treatment, the oxygenation index and PaCO_2_ index in the observation group were better than those in the control group (*P* < 0.05). The results are shown in Table 1.

**Table 1:** Comparison of oxygenation and PaCO_2_ before and after treatment between the two groups

Group	Oxygenation index		PaCO_2_	
	Treatment 1d	Treatment 7d	Treatment 1d	Treatment 7d
Control group (*n* = 19)	108.05 ± 12.41	168.41 ± 8.89	50.78 ± 5.92	39.59 ± 4.09
Observation group (*n* = 19)	107.98 ± 11.27	210.92 ± 13.54	47.18 ± 5.21	40.50 ± 4.59
t value	0.018	11.442	1.990	0.725
P value	0.986	0.000	0.054	0.473

### Comparison of PCT and IL-6 before and after treatment between the two groups

After treatment, the levels of PCT and IL-6 indexes in the observation group were better than those index in the control group (*P* < 0.05). The results are shown in Tables 2 and 3.

**Table 2:** Comparison of PCT before and after treatment between the two groups

Group	PCT	
	Treatment 1d	Treatment 7d
Control group (*n* = 19)	0.70 ± 0.77	0.11 ± 0.44
Observation group (*n* = 19)	0.88 ± 0.61	0.09 ± 0.29
t value	0.798	2.177
P value	0.430	0.036

**Table 3:** Comparison of IL-6 before and after treatment between the two groups

Group	IL-6	
	Treatment 1d	Treatment 7d
Control group (*n* = 19)	63.18 ± 14.99	22.49 ± 9.79
Observation group (*n* = 19)	62.92 ± 12.85	15.56 ± 4.43
t value	0.056	2.809
P value	0.956	0.009

### Comparison of chest X-ray before and after treatment between the two groups

As shown in Table 4. The changes of chest X-ray before and after treatment were compared between the two groups, and the clinical efficacy of the observation group before and after treatment was better than that of the control group (*P* < 0.05).

**Table 4:** Comparison of chest X-ray before and after treatment between the two groups

Group	Effective	Ineffective	P value
Control group (*n* = 19)	5(26.3)	14(73.7)	0.049
Observation group (*n* = 19)	12(63.2)	7(36.8)	

## Discussion

COVID-19 is a serious threat to the lives and health of people around the world, and a large number of scholars and experts have made great contributions to the treatment of COVID-19. Previous studies have shown that Autotaxin can be used as a therapeutic target for COVID-19 [17], in addition, baricitinib and Tocilizumab are effective in the treatment of COVID-19 [18, 19]. And, there are also reports on the after-effects of COVID-19 [20, 21]. However, in the clinical diagnosis and treatment of patients, we found that the lungs of patients with severe novel coronavirus infection were often accompanied by poor deep sputum drainage [22]. And bed rest and sedation drugs would aggravate the weakness of the diaphragm and lead to worsening respiratory symptoms, and even cause serious sequelae [23].. So, therapies that change the patient’s position and breathing patterns may help patients recover, but this has not been studied. Therefore, this paper chooses two kinds of respiratory rehabilitation therapy to study to complement the research gap in this field.

By changing the patient’s position, the prone position can re-expand the collapsed alveoli to improve the ventilation and blood flow ratio in the lung gravity-dependent area, reduce respiratory dead space, improve the movement pattern and position of the diaphragm, Improves diaphragm movement pattern and position, thereby improving oxygenation and airway clearance [24]. And, it can also reduce the occurrence of lung injury, reduce the secondary multiple organ dysfunction and mortality caused by oxygenation disorders in patients [25].

In prone position, combined airway clearance technology can be used to release and drain sputum through postural drainage or thoracic vibration to improve the efficiency of mucus removal [26]. Using the bidirectional air flow mechanism, the manual training technique intervenes between exhalation and inhalation, releases the thorax, increases the degree of movement of the thorax and diaphragm, increases lung compliance and elastic retraction force. And performs ventilation and perfusion in more lung segments, effectively improving blood gas indexes and respiratory function. The results of this study showed that the indexes of the observation group after airway clearance technique combined with prone ventilation were better than those of the control group (*P* < 0.05), This study further confirmed the effect of airway clearance technique combined with prone position ventilation.

Patients with COVID-19 often have deep sputum, poor sputum drainage and obstruction of small airways lead to chronic infection and aggravation of inflammation [27]. With the release of contents of accumulated neutrophils, the viscosity of mucus further increases, thus forming a vicious cycle of obstruction-infection-inflammation [28].. With prolonged bed immobilization, hospitalized patients with COVID-19 are at increased risk of developing a second bacterial infection [29]. Prone lung ventilation treatment combined with airway clearance technology, slow release of thick sputum, effectively promote deep sputum drainage and reduce the risk of co-infection [30]. During the training process of airway clearance manipulation, the patient’s exercise volume increased, which stimulated the cerebral cortex, regulated the neurohumoral system, improved the body’s stress ability, regulated immune function, and alleviated systemic inflammatory response [31, 32]. Therefore, airway clearance technology combined with prone ventilation therapy can help improve the changes of inflammatory indicators in patients with severe COVID-19. The results of this study showed that airway clearance technology combined with prone ventilation therapy had a positive effect on improving the changes of inflammatory indicators in patients with severe COVID-19 (*P* < 0.05).

The results showed that the combination of airway cleaning technique and prone position ventilation can effectively relieve pulmonary infection, and effective removal of lung secretions. It can improve the total effective rate and oxygenation index of patients and improve the respiratory function and clinical symptoms of patients. Therefore, clinical study data show that airway clearance technology combined with prone ventilation is safe and effective, and can be widely applied in the treatment of COVID-19(severe) patients.

In addition, there are some limitations in this study, such as small sample size and limited follow-up time after discharge. These limitations will be further discussed and explored in subsequent studies.

## Data Availability

The datasets generated and analyzed during the current study are not publicly available due [PROTECT PATIENT PRIVACY] but are available from the corresponding author on reasonable request.
